# Intrafraction pancreatic tumor motion patterns during ungated magnetic resonance guided radiotherapy with an abdominal corset

**DOI:** 10.1016/j.phro.2021.12.001

**Published:** 2021-12-21

**Authors:** Guus Grimbergen, Hidde Eijkelenkamp, Hanne D. Heerkens, Bas W. Raaymakers, Martijn P.W. Intven, Gert J. Meijer

**Affiliations:** Department of Radiation Oncology, University Medical Center Utrecht, the Netherlands

**Keywords:** Pancreatic cancer, Intrafraction motion, MRgRT, SBRT, Motion management

## Abstract

**Background:**

Stereotactic body radiotherapy (SBRT) has been shown to be a promising therapy for unresectable pancreatic tumors. However, intrafraction motion, caused by respiratory motion and organ drift, is one of the main concerns for efficient dose delivery in ungated upper abdominal radiotherapy. The aim of this study was to analyze the intrafraction gross tumor volume (GTV) motion in a clinical cohort.

**Materials and methods:**

We included 13 patients that underwent online adaptive magnetic resonance (MR)-guided SBRT for malignancies in the pancreatic region (5 × 8 Gy). An abdominal corset was fitted in order to reduce the abdominal respiratory motion. Coronal and sagittal cine magnetic resonance images of the tumor region were made at 2 Hz during the entire beam-on time of each fraction. We used deformable image registration to obtain GTV motion profiles in all three directions, which were subsequently high-pass and low-pass filtered to isolate the motion caused by respiratory motion and baseline drift, respectively.

**Results:**

The mean (SD) respiratory amplitudes were 4.2 (1.9) mm cranio-caudal (CC), 2.3 (1.1) mm ventral-dorsal (AP) and 1.4 (0.6) mm left–right (LR), with low variability within patients. The mean (SD) maximum baseline drifts were 1.2 (1.1) mm CC, 0.5 (0.4) mm AP and 0.5 (0.3) mm LR. The mean (SD) minimum baseline drifts were −0.7 (0.5) mm CC, −0.6 (0.5) mm AP and −0.5 (0.4) mm LR.

**Conclusion:**

Overall tumor motion during treatment was small and interfractionally stable. These findings show that high-precision ungated MR-guided SBRT is feasible with an abdominal corset.

## 1 Introduction

Pancreatic cancer is one of the most aggressive cancer types, with a median overall survival rate of typically around 19 months. For non-metastatic unresectable tumors or isolated local recurrences, prognosis is around 14–15 months [1], [2]. Stereotactic body radiotherapy (SBRT) has been shown to be a promising therapy for these tumor types in terms of local disease control [3], [4], [5], [6]. The main challenge in SBRT in the upper-abdomen is avoiding the many radiosensitive gastro-intestinal organs, such as the duodenum, small bowel, colon, stomach or post-resection anastomoses, which often lie in close proximity to the gross tumor volume (GTV). The introduction of magnetic resonance guided radiotherapy (MRgRT) allows for online plan adaptation based on magnetic resonance imaging (MRI) visualized anatomy at each fraction. The superior soft tissue contrast of MRI, together with the capability for replanning at each fraction, enables the delivery of higher biological effective doses in a lower number of fractions [7], [8].

However, intrafraction motion remains of concern for ablative treatments with SBRT. Early studies on respiratory intrafraction motion have found GTV motion to be large with average cranio-caudal motion amplitudes of over 20 mm, and highly variable with standard deviations of over 15 mm [9], [10]. Consequently, research has mainly been focused on gating schemes [11], [12] or development of motion surrogate models [13], [14]. Alternatively, the use of abdominal compression techniques during treatment, *e.g.* with an abdominal corset, can reduce the respiratory motion in the abdominal area, thereby significantly reducing the residual cranio-caudal motion in the upper abdominal structures [15].

Besides respiratory motion, dosimetric inaccuracies can also be caused by gastro-intestinal (GI) organs slowly drifting away from the planning position during treatment, for example due to bowel peristalsis or muscle relaxation or tensioning. Previous research on abdominal organ drifts during treatment has reported relatively small deviations of 1–4 mm from the baseline [16], [17]. However, this type of motion is often assumed to be non-periodic and linear causing a dose shift and may therefore be more of concern than periodic respiratory motion, which only results in a blurring of the dose.

The aim of this study was to retrospectively analyze and characterize respiration and drift motion patterns of pancreatic tumors in our currently treated patient population, across a clinical cohort of 13 patients that underwent MRgRT.

## 2 Materials & methods

### 2.1 Patients

Thirteen patients with malignancies in the pancreatic region underwent MR-guided SBRT between February 2020 and September 2020. The cohort included seven male and six female patients, with a median age of 64 years (range 40–76 years). Consecutive informed consent was provided for registry in the prospective Multi-OutcoMe EvaluatioN of radiation Therapy Using the MR-linac (MOMENTUM) study (NCT04075305), which has been approved by the institutional review board (IRB) of the University Medical Center Utrecht.

A summary of the patient and treatment characteristics is given in Table 1. Detailed characteristics for the individual patients are given in Supplementary material A.

**Table 1:** Patient and treatment characteristics.

	*n* = 13 (%)
Sex	
Male	7 (54)
Female	6 (46)

Age (years)	
Median (IQR)	64 (53–69)
range	40–76

Tumor volume (cm^3^)	
GTV	
median (IQR)	26 (15–62)
range	13–108
PTV	
median (IQR)	49 (34–62)
range	25–159

Diagnosis	
Pancreatic adenocarcinoma	
Locally advanced	4 (31)
Locally recurrent	4 (31)
Cholangiocarcinoma	
Locally recurrent	4 (31)
Adrenocortical carcinoma	
Locally recurrent	1 (8)

Prior treatment	
Surgery	9 (69)
Time prior to SBRT (months)	
median (IQR)	15 (12–19)
Range	9–25

### 2.2 Image acquisition and treatment protocol

Before treatment, each patient was provided with a polyurethane Neofrakt abdominal corset (Spronken Orthopedie NV, Genk, Belgium) [15]. This is a custom fitted corset that was molded to the patient’s lumbar spine, directly after their first consult with the attending radiation oncologist. The molding takes around 20 min. Two to three weeks prior to the start of treatment, each patient underwent a planning and simulation session. First, the patient underwent computed tomography (CT) scanning on a Philips Brilliance big bore CT scanner (Philips BV, Best, the Netherlands), during which the corset was applied and fastened with Velcro straps. The tightness of the straps was marked with a pen to ensure reproducibility. Immediately afterwards, the patient was scanned on a 1.5 T Philips Ingenia MRI scanner (Philips BV, Best, the Netherlands). During this session, a 3D *T*_2_-weighted (T2w) scan (field of view (FOV): 451 × 451 × 220 mm, voxel size: 0.64 × 0.64 × 2.00 mm, echo time/repetition time (TE/TR): 124/1300 ms, flip angle (FA): 90°) was acquired, as well as gadolinium-enhanced coronal (FOV: 450 × 450 mm, voxel size: 2.01 × 2.01 × 7.00 mm, TE/TR: 1.31/2.62 ms, FA: 50°) and sagittal (FOV: 320 × 320 mm, voxel size: 1.43 × 1.43 × 7.00 mm, TE/TR: 1.43/2.86 ms, FA: 50°) balanced fast field echo (bFFE) cine MRIs during one minute at 2 Hz. Treatment was carried out on the Elekta Unity (Elekta AB, Stockholm, Sweden) Magnetic Resonance Linear Accelerator (MR-Linac), a 7 MV linear accelerator combined with a 1.5 T wide bore MRI scanner. The SBRT treatment itself consisted of five fractions of 8 Gy, given every other weekday. The protocol for each fraction was as follows. First, the patient was positioned on the MR-Linac table on a custom fitted vacuum cushion, and secured with the abdominal corset. Next, a 3D T2w scan was acquired. The GTV and organs at risk (OAR) were contoured on this scan by the attending radiation oncologist. We employed an isotropic GTV to planning target volume (PTV) margin of 3 mm. During contouring, a position verification (PV) 3D T2w scan was acquired, to ensure no large position shifts had occurred. After contouring, a 9–14 beam intensity-modulated radiotherapy (IMRT) plan was generated and the treatment delivery was started after approval by the physician. During the entire beam-on time, interleaved coronal and sagittal (FOV: 426 × 426 mm, voxel size: 1.06 × 1.06 × 7.00 mm, TE/TR: 1.37/2.73 ms, FA: 40°) bFFE cine MRIs were acquired at 2 Hz. The planes were centered on the GTV. Ultimately, once irradiation had been completed, a final 3D T2w post scan was acquired to evaluate the 3D anatomy of the patient at the end of the treatment session.

**Fig. 1:**
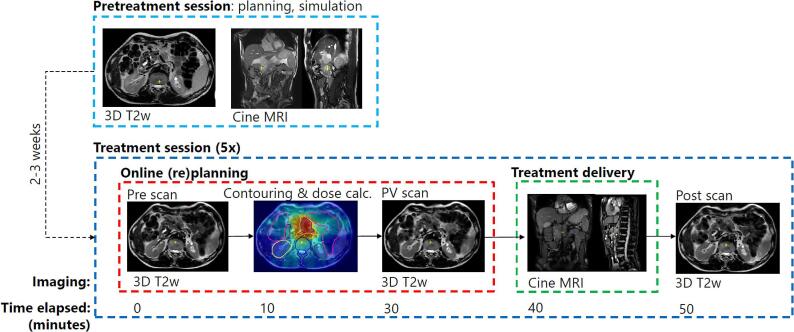
The workflow of the treatment protocol, with the average time elapsed in minutes at each step of the online adaptive workflow.

An illustration of the treatment workflow is given in Fig. 1.

### 2.3 Motion analysis

The continuous interleaved sagittal and coronal cine MRIs during the entire beam-on time allowed for the extraction of high-temporal resolution 3D motion profiles of the GTV from start to end of treatment.

All computations were performed in Matlab 2019a (Mathworks Inc., Natick, MA, USA). As non-rigid registration algorithm, we employed the GPU implementation of EVolution [18]. The dynamics within a set of cine MRIs were deformably registered to the 10th dynamic of that set. The 10th dynamic was chosen to ensure sufficient contrast has been manifested for successful image registration. Then, the GTV contour, as delineated on the pre 3D MRI, was propagated onto the coronal and sagittal planes of the 10th dynamic. In case of the planning cine MRIs, for which no direct delineation of that day was available, the contour of the first fraction was used. The contour was manually translated to match the tumor position in the image, and converted to a binary mask. From there, the deformation vector fields (DVFs) from the image registration were used to warp the GTV masks from the 10th dynamic to all other dynamics, creating a tumor delineation that moved along with the anatomy in 2D + time in both the sagittal and coronal plane. We subsequently tracked the displacement of the GTV centroid over time to obtain the motion profiles. The displacement position was normalized to the average position of the first 30 s, which served as the reference starting position of the GTV during treatment.

Cranio-caudal motion profiles were obtained from both the coronal and sagittal cine MRIs, denoted as *CC_C_* and *CC_S_* respectively. Left-right (*LR*) motion was obtained from the coronal plane and anterior-posterior (*AP*) motion from the sagittal plane.

Since the motion profiles contain signals from both respiratory and drift motion, both had to be isolated to ensure reliable results. To analyze the respiratory motion, the raw motion profiles were high-pass filtered with an elliptic infinite impulse response (IIR) filter. Visual inspection of the average power spectrum of all motion profiles showed that the respiratory frequency components were between 0.10 and 0.25 Hz (see Supplementary material B). From this, a cutoff frequency of 0.05 Hz was chosen for the high-pass filter, which should be high enough to remove all low-frequency motion due to drift, but low enough to avoid attenuation of the respiratory signal. The drift motion was obtained by low-pass filtering the raw signal with a moving average filter with a sliding window of 50 samples, or 25 s. The duration of cine MRIs from the simulation session was deemed too short to reliably extract data on baseline drift, and only respiratory motion was analyzed for these sets.

In all four displacement directions, we calculated the respiratory amplitude *M*_resp_ as the difference between the maximum and minimum value in the high-pass filtered signal, excluding the top and bottom 5 percentiles to reduce sensitivity to outliers. The baseline drift extrema *M*_drift_*_,_*_min_ and *M*_drift_*_,_*_max_ are calculated as the minimum and maximum values in the low-pass filtered signal, respectively.

Since motion in the cranio-caudal direction was measured in both the coronal and sagittal plane, we performed Bland-Altman analysis to assess the agreement between the two methods of measurement for cranio-caudal respiration amplitude and minimum and maximum baseline drift.

## 3 Results

### 3.1 Motion analysis

**Fig. 2:**
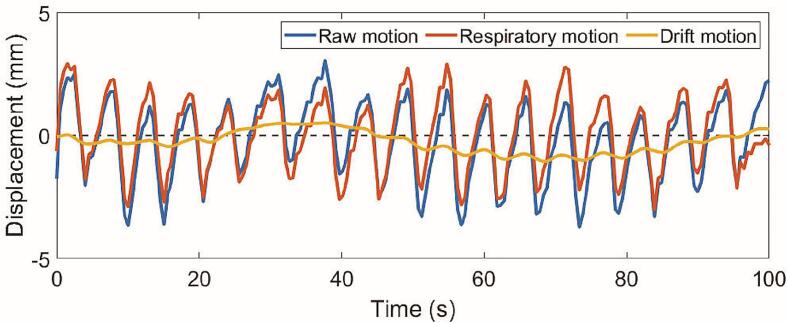
The first 100 s of an exemplary *CC_C_* motion signal that has been both high-pass and low-pass filtered to obtain the isolated respiratory and drift motion. Note that the respiratory signal has a stable equilibrium around *y* = 0, while its amplitude remains equal to the raw signal.

An example of the respiratory and drift motion decoupled from a raw *CC_C_* motion profile is given in Fig. 2. The two frequency components were clearly visible in the raw motion signal. The low-pass filtered respiratory motion had a stable equilibrium around the baseline, with a peak-to-peak amplitude equal to the unfiltered signal. The high-pass filtered drift motion lacked any high frequency oscillations and follows the average position of the raw motion profile. These findings indicate a successful filtering strategy.

### 3.2 Respiratory motion

**Fig. 3:**
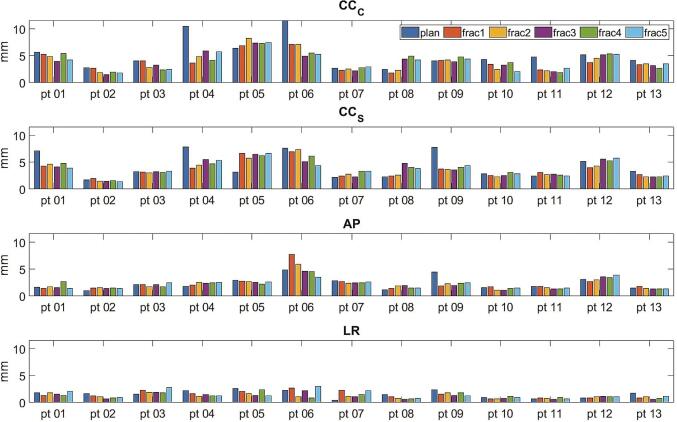
The peak-to-peak respiratory amplitudes, excluding the top and bottom 5th percentiles, for all patients and all fractions.

The mean (SD) respiratory amplitudes over all patients were 4.2 (1.9) mm *CC_C_*, 3.9 (1.7) mm *CC_S_*, 2.3 (1.1) mm *AP* and 1.4 (0.6) mm *LR* (see Table 2). The respiratory amplitudes for individual fractions, in each direction, are plotted in Fig. 3. Overall, there was a good agreement between the *CC* values extracted from the coronal and sagittal cine MRIs. Bland-Altman analysis on the measurement differences between *CC_C_* and *CC_S_* yielded a 95% CI of −1.68 – 2.29 mm. The mean difference was 0.3 mm, indicating slightly higher amplitude for the coronal cine MRIs (Supplementary material C).

**Table 2:** Mean and SD of the respiratory amplitudes and minimum and maximum baseline drifts over all patients and fractions.

	*M*_resp_ mean (SD) – mm	*M*_drift,min_ mean (SD) – mm	*M*_drift,max_ mean (SD) – mm
*CC_C_*	4.2 (1.9)	−0.7 (0.5)	1.2 (1.1)
*CC_S_*	3.9 (1.7)	−0.7 (0.5)	1.2 (1.0)
*AP*	2.3 (1.1)	−0.6 (0.5)	0.5 (0.4)
*LR*	1.4 (0.6)	−0.5 (0.4)	0.5 (0.3)

There was a large similarity of the respiration amplitude within individual patients for the different fractions. For patients 01, 04, 06, 09 and 11, we observed that the respiratory amplitude during the planning session was higher than during to the treatment fractions for either the sagittal and/or coronal cine MRI assessment.

### 3.3 Baseline drift

**Fig. 4:**
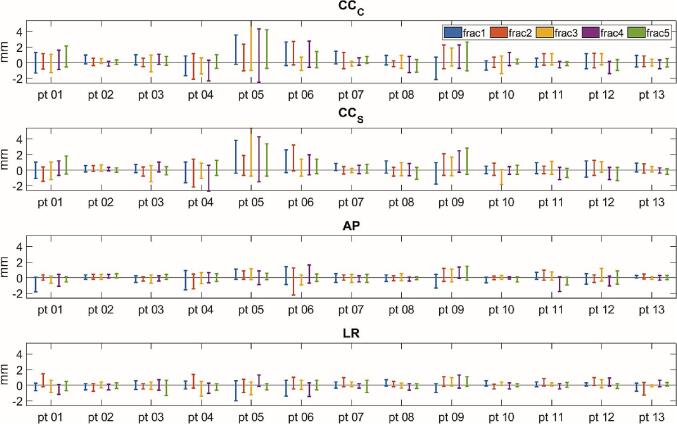
The maximum and minimum baseline drifts for all patients and all fractions. In each bar, the top point indicates the maximum drift value and the bottom point indicates the minimum value.

The mean (SD) minimum baseline drifts over all patients and fractions were −0.7 (0.5) mm *CC_C_*, −0.7 (0.5) mm *CC_S_*, −0.6 (0.5) mm *AP* and −0.5 (0.4) mm *LR*. The mean (SD) maximum baseline drifts were 1.2 (1.1) mm *CC_C_*, 1.2 (1.0) mm *CC_S_*, 0.5 (0.4) mm *AP* and 0.5 (0.3) mm *LR* (see Table 2). The minimum and maximum baseline drifts for all individual fractions are plotted in Fig. 4. The displacements were generally the largest in cranio-caudal direction. There was again a good agreement between *CC_C_* and *CC_S_* for most patients: for *M*_drift_*_,_*_min_, the 95% CI of the *CC_C_* – *CC_S_* differences was −0.61 – 0.54 mm and for *M*_drift_*_,_*_min_, this was −0.60 to 0.75 mm. In both cases, there was a negligible bias of −0.03 mm and 0.07 mm, respectively (Supplementary material C).

Overall, the values were smaller than the respiratory amplitudes. The patients with the largest differences between *M*_drift_*_,_*_min_ and *M*_drift_*_,_*_max_, patients 04, 05, 06 and 09 were characterized by a systematic unidirectional drift. In most of these patients where there was a systematic drift observed, the tumor moved in the cranial direction. For seven patients, the drifts were much smaller with the difference between *M*_drift_*_,_*_min_ and *M*_drift_*_,_*_max_ less than 2 mm, and their absolute values within 1 mm of each other.

## 4 Discussion

This study provided an analysis of pancreatic tumor motion during online MR-guided SBRT for patients wearing an abdominal corset. In our cohort, respiratory amplitudes were small and stable within patients. Drift motions were small as well, with no large GTV displacements observed during the treatments. Wearing of the corset was tolerated well, and none of the patients reported pain or other discomfort that urged loosening the corset.

Based on the van Herk margin recipe [19], [20], the observed intrafraction motion would require margins of 1.95 mm *CC*, 1.10 mm *AP*, and 0.60 mm *LR* to obtain adequate dose coverage (Supplementary material D). This is well below the 3 mm isotropic PTV margins used in this study. Moreover, these margins were less than 1.0 mm larger compared to the margins in a complete static configuration. This substantiates our hypothesis that additional motion mitigation techniques (*e.g* gating or tracking) are of limited value when using an abdominal corset.

The respiratory displacements observed in this study are substantially smaller than early literature, with mean cranio-caudal amplitudes of under 5 mm [9], [10]. We believe this can mainly be attributed to the use of the abdominal corset. Remarkably, the original study on this corset from Heerkens et al. also found higher motion amplitudes than in this study, even with the corset employed (6.0 mm *CC_C_*, 5.6 mm *CC_S_*, 2.6 mm *AP* and 2.2 mm *LR*) [15]. It should be noted that Heerkens et al. analyzed the amplitudes of the raw motion profiles without decoupling respiration and drift, resulting in larger values. Secondly, the authors excluded the top and bottom 2.5% of the values, while our study excluded the top and bottom 5%. Thirdly, both our study and Heerkens et al. included a small number of patients: 13 and 10 patients respectively. The differences in results could be an effect due to these small cohort sizes. Finally, Heerkens et al. analyzed mainly locally advanced tumors. Our patient cohort included mainly recurrent carcinomas, often restricted by fibrotic tissue, which can further inhibit tumor mobility. However, due to our small population size and imbalance of recurrent and advanced carcinomas within our cohort, we can provide no conclusive evidence for a connection between tumor mobility versus site and stage. A larger and more balanced patient population might lead to more insights into this differentiation.

One of our main findings was that within patients, there was little interfractional variability of respiratory amplitudes. Given that each patient appears to possess a characteristic amplitude, this could lead to an expansion of the online adaptive workflow. As an example, PTV margins can be tailored to the respiration amplitude of the patient.

For patients 01, 04, 06, 09 and 11, we observed that the respiratory amplitude during the planning session was higher than during to the treatment fractions for either the sagittal and/or coronal cine MRI assessment. Additionally, we also noted larger differences between the values for *CC_C_* and *CC_S_* in these cases. These discrepancies might have several causes, but the most likely explanation is that the corset could have been fastened less tightly during the planning sessions than during treatment. The setting of the straps with which the corset was fastened was marked during the CT planning session. However, it might occurred that during the subsequent MRI session immediately afterwards, the corset was not fastened all the way to the marks. While corset tightness is not as clinically relevant during the planning session as during treatment, it would have been a proper procedure to ensure equal compression during all sessions. Nonetheless, it further supports our theory that a properly tightened corset, while also mitigating motion in general, would lead to less variability in motion amplitudes within patients.

The more prominent differences between the *CC_C_* and *CC_S_* amplitude assessments could well be associated to possible misalignments of sagittal and coronal planes on the planning cine MRIs, as these planes were aligned by the radiographers in the absence of the GTV definition. Depending on GTV shape, through plane motion in the 2D cine MRIs could also have led to an over- or underestimation of *CC_C_* and *CC_S_* amplitudes.

Motion assessments due to slow organ drift revealed only small extrema with respect to the baseline. Moreover, drift did not occur in a linear fashion, where the average GTV position would slowly deviate away from the baseline, but rather inhibited its own periodic motion. Any large deviations often resolved back towards initial position, and in eight out of thirteen patients the values in Fig. 4 show approximately equally large drifts in all three directions. This corresponds to the findings of Cusumano et al. [16], which reported no large drifts in the pancreas subgroup of their multi-site cohort.

The use of orthogonal 2D planes poses some limitations. First, the planes were centered on the GTV, and as a result, most of the surrounding OARs fell out of plane and could not reliably be tracked. The intrafraction motion of the many radiosensitive OARs is valuable information for determining whether the treatment was delivered safely. Upon visual basis it was determined that in close proximity to the GTV, the surrounding tissue moved quasi-rigidly along with the GTV. Volumetric imaging would have provided the necessary information to track the OARs as well, but this is much slower than 2D cine MRI. Second, we defined the GTV motion as the displacement of its center of mass over time, not accounting for deformations internally or at the boundary. However, visual inspection of the cine MRIs again yielded that the tumor itself did not appear to deform significantly from physiological motion. Therefore, we believe that the position of its center of mass was adequate to provide an accurate motion profile.

In conclusion, ungated, high-precision MR-guided SBRT for pancreatic tumors is feasible for patients that wear an abdominal corset, potentially allowing dose escalation strategies. Motion mitigation using corsets could well be a simple and attractive alternative for more complex gating and tracking approaches when treating abdominal lesions.

## Declaration of Competing Interest

The authors declare that they have no known competing financial interests or personal relationships that could have appeared to influence the work reported in this paper.
