# Supplementary material for: Intrafraction pancreatic tumor motion patterns during ungated magnetic resonance guided radiotherapy with an abdominal corset
Source: Phys Imaging Radiat Oncol. 2021 Dec 21;21:1–5. doi: 10.1016/j.phro.2021.12.001 (PMC8715205; doi:10.1016/j.phro.2021.12.001)
Supplement: Supplementary data 1 [file mmc1.docx]

# Supplementary material A: Detailed patient characteristics

Table A: Individual patient characteristics. L(A/R)PC: Locally (advanced/recurrent) pancreatic carcinoma. LRCC: Locally recurrent cholangiocarcinoma. *Not applicable for recurrent carcinomas.

|  | **Age** | **Sex** | **GTV/PTV**  **volume** (cm^3^) | **Diagnosis** | **Pancreas tumor**  **location*** | **Prior**  **Histology treatment** | |
| --- | --- | --- | --- | --- | --- | --- | --- |
| pt 01 | 65 | M | 102/154 | LAPC | Corpus | None Adenocarcinoma | |
| pt 02 | 57 | F | 20/39 | LRCC | *n.a.* | Distal Surgery, 15  Cholangiocarcinoma months prior | |
| pt 03 | 64 | F | 14/35 | LRCC | *n.a.* | Distal Surgery, 19  Cholangiocarcinoma months prior | |
| pt 04 | 40 | M | 62/105 | LAPC | *n.a.* | Ampullary carcinoma | None |
| pt 05 | 57 | M | 108/159 | LAPC | Head | Adenocarcinoma | None |
| pt 06 | 69 | M | 26/49 | LRPC | *n.a.* | Ductal adenocarcinoma | Surgery, 14 months prior |
| pt 07 | 53 | M | 13/25 | LRPC | *n.a.* | Colloid carcinoma | Surgery, 24 months prior |
| pt 08 | 75 | F | 96/146 | LAPC | Corpus | Unknown | None |
| pt 09 | 64 | M | 18/34 | LRPC | *n.a.* | Ductal adenocarcinoma | Surgery, 9 months prior |
| pt 10 | 41 | F | 27/53 | LRPC | *n.a.* | Ductal adenocarcinoma | Surgery, 14 months prior |
| pt 11 | 47 | M | 15/29 | Recurrent adrenocortical carcinoma | *n.a.* | Adrenocortical carcinoma | Surgery, 25 months prior |

Surgery, 18

pt 12 76 M 52/84 LRCC *n.a.*

Adenocarcinoma months prior

| pt 13 | 75 | F | 13/25 | LRCC | *n.a.* | Adenocarcinoma ductus choledochus | Surgery, 12 months prior |
| --- | --- | --- | --- | --- | --- | --- | --- |

# Supplementary material B: Average motion profile power spectrum


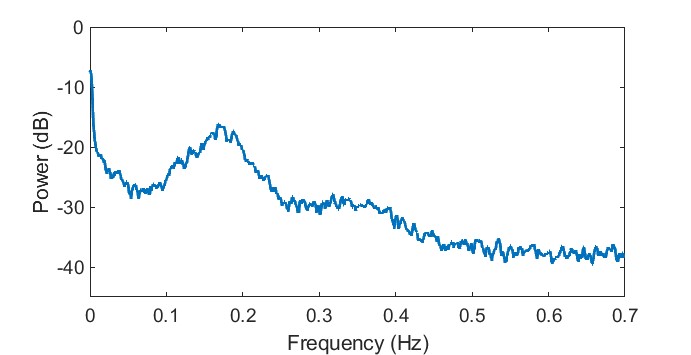


Figure B: Average power spectrum of all motion profiles. The frequency components of the respiratory signals fall between 0.10 and 0.25 Hz.

# Supplementary material C: Bland-Altman plots of *CC_C_* and *CC_S_* measurements


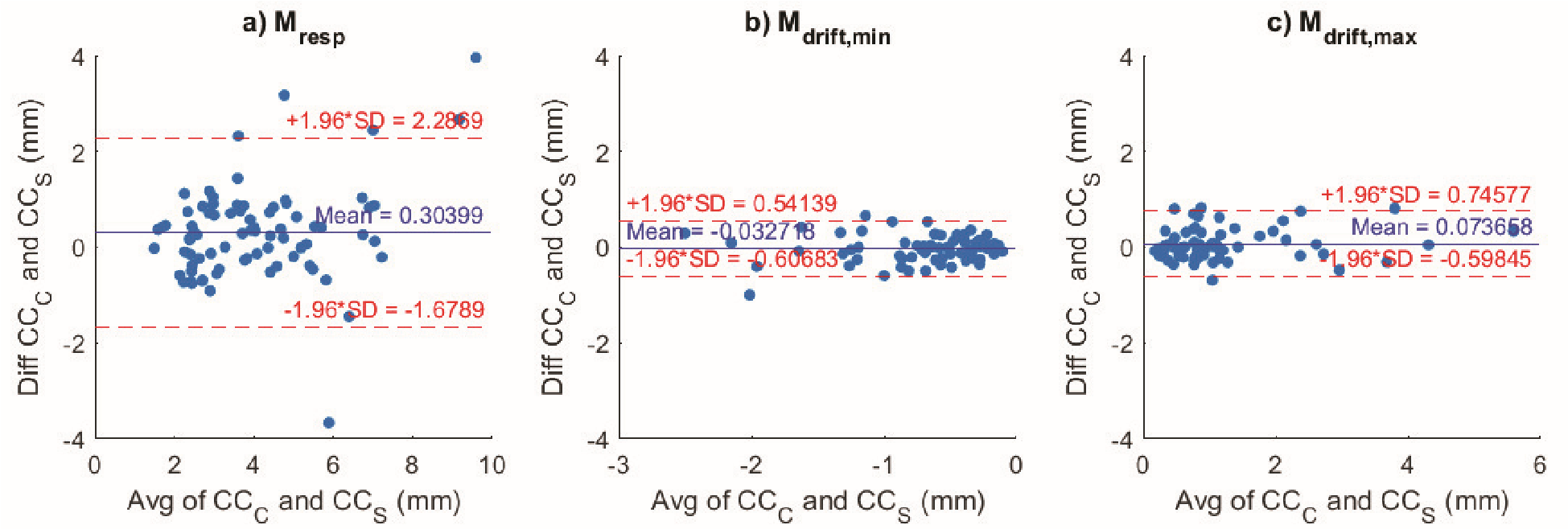


Figure C: Bland-Altman plots of the *CC_C_* and *CC_S_* measurements: a) the respiratory amplitudes; b) the minimum baseline drift; c) the maximum baseline drift.

# Supplementary material D: Margin assessment

Van Herk et al. derived a nonlinear, probabilistic model for PTV margin calculation, taking into account the systematic ($\Sigma$) and random ($\sigma$) errors [19,20]. These errors are both defined as any error leading to a deviation from the prescribed dose to delivered dose. Systematic errors characterize errors in treatment setup, and random errors characterize errors in treatment execution. Analytically, the required margin to deliver at least 95% of the prescribed dose to the GTV in 90% of the patients is:

$$m=2.5\Sigma+1.64\left( \sigma-\sigma_{p} \right)$$

where $\sigma^{2}=\sigma_{m}^{2}+\sigma_{p}^{2}$ is the total SD of all random errors: GTV drift motion $\sigma_{m}$ and beam penumbra width $\sigma_{p}$. We can further incorporate the SD of the respiratory motion $\sigma_{r}$, by adding this quadrature to $\sigma$. We reported the SDs of the respiratory motion in section 3.1.1. This yields the following equation:

$$\boldsymbol{m}=2.5\boldsymbol{\Sigma+}1.64(\sqrt{\boldsymbol{\sigma}_{m}^{2}+\boldsymbol{\sigma}_{r}^{2}+\boldsymbol{\sigma}_{p}^{2}}-\boldsymbol{\sigma}_{p})$$

Note that we now define the variables as vectors, such that we can have different values for all three principle directions of movement, and thus anisotropic margins.

As measurements for the systematic and random errors, we take the average value of each motion profile per patient and per fraction. Since we have set a displacement of 0 to the midposition, any average deviation will lead to treatment inaccuracies. For each patient, we calculate the mean and SD error over all five fractions. From these means, we calculate $\boldsymbol{\Sigma}$ as the SD over all patients. From the SDs, we calculate $\boldsymbol{\sigma}_{m}$ as the root-mean-square over all patients.

We set $\boldsymbol{\sigma}_{p}$ to 3 mm isotropic. We calculate the PTV margins for two cases: $\boldsymbol{\sigma}_{r}$= 0 and $\boldsymbol{\sigma}_{r}$ = $\boldsymbol{\sigma}_{r}$, to compare how much extra margin is needed to account for respiratory motion on top of the remaining treatment setup and execution errors. In total, this yields the margins requirements as presented in Table D.1. These values indicate that when accounting for the respiratory amplitudes found in our cohort, we need additional PTV margins of 0.90 mm *CC*, 0.32 mm *AP*, and 0.10 mm *LR*. The total required margins of 1.95 mm *CC*, 0.82 mm *AP*, and 0.44 mm *LR* are well below the 3 mm isotropic margins that we used during treatment. This adds to our conclusion that with the observed intrafraction motion, ungated SBRT can be delivered efficiently, with adequate target coverage.

Table D: Systematic and random errors, respiratory SDs, and required margins according to the van Herke margin recipe. All in mm.

|  | *CC* | *AP* | *LR* |
| --- | --- | --- | --- |
| $\boldsymbol{\Sigma}$ | 0.40 | 0.19 | 0.13 |
| $\boldsymbol{\sigma}_{m}$ | 0.39 | 0.27 | 0.28 |
| $\boldsymbol{\sigma}_{r}$ | 1.90 | 1.10 | 0.06 |
| Margin, $\boldsymbol{\sigma}_{r}=\boldsymbol{0}$ | 1.05 | 0.50 | 0.34 |
| Margin, $\boldsymbol{\sigma}_{r}=\boldsymbol{\sigma}_{r}$ | 1.95 | 0.82 | 0.44 |
